# Risk Factors Associated With Major Skin Diseases: A Cross‐Sectional Study From Bangladesh

**DOI:** 10.1155/ghe3/8995557

**Published:** 2026-06-20

**Authors:** Md. Abrar Ashfaq Khan, Md. Rashed Babu, Mohammad Ohid Ullah

**Affiliations:** ^1^ Department of Data Science and Statistics, Oregon State University, Corvallis, Oregon, USA, oregon.gov; ^2^ Department of Statistics, Shahjalal University of Science and Technology, Sylhet, Bangladesh, sust.edu

**Keywords:** acne vulgaris, Bangladesh, environmental factors, fungal infection, heritable factors, principal component analysis, skin disease

## Abstract

**Background:**

Skin diseases are prevalent in Bangladesh, affecting individuals across all age groups and significantly impacting both personal and community health.

**Objective:**

This study aims to identify risk factors associated with certain skin diseases in Bangladesh, specifically examining lifestyle, environmental, food‐related, and heritable factors.

**Methodology:**

A cross‐sectional study was conducted across three major divisions in Bangladesh (Dhaka, Rangpur, and Chattogram) from December 2022 to July 2023. Data were collected from 788 respondents using a structured questionnaire through a purposive quota sampling technique. The questionnaire covered self‐reported skin disease status, sociodemographic characteristics, and potential risk factors. The analysis employed univariate tools, principal component analysis (PCA) to reduce correlated risk factors into uncorrelated components, and principal component logistic regression (PCLR) to assess associations. Goodness‐of‐fit testing was conducted to validate model calibration, and sensitivity analyses adjusting for sociodemographic confounders were performed to confirm the robustness of identified associations.

**Results:**

Among the 788 respondents, 61.80% (*n* = 487) reported skin diseases. Among these, atopic dermatitis was the most prevalent condition (72.07%), followed by fungal infection (26.90%), acne vulgaris (20.74%), seborrheic dermatitis (13.55%), and psoriasis (8.21%); as multiple conditions could be reported concurrently, these percentages are not mutually exclusive. Through PCA and PCLR analyses, several associations were initially identified. Following rigorous goodness‐of‐fit testing, two associations demonstrated adequate model calibration: heritable factors were significantly associated with fungal infection, and environmental factors were significantly associated with acne vulgaris. A one‐unit increase in heritable factors (for instance, having an additional family member with skin disease) increased the odds of fungal infection by 34% in unadjusted models (OR: 1.34, 95% CI: 1.08–1.66), and a one‐unit increase in environmental factors increased the odds of acne vulgaris by 45% in unadjusted models (OR: 1.45, 95% CI: 1.16–1.80). After adjusting for age, sex, division, and disease‐specific confounders, these associations remained significant (adjusted OR: 1.37, 95% CI: 1.09–1.74 for fungal infection; adjusted OR: 1.32, 95% CI: 1.05–1.67 for acne vulgaris), confirming their robustness.

**Conclusion:**

This study provides valuable insights into risk factors associated with skin diseases in Bangladesh. The findings highlight that family history plays a significant role in fungal infection susceptibility, while environmental exposures are important determinants of acne vulgaris. The study underscores the importance of rigorous goodness‐of‐fit testing and confounder adjustment in observational research, as several initially significant associations did not demonstrate adequate model calibration or lost significance after adjustment. These validated findings suggest the need for targeted interventions addressing heritable predisposition for fungal infections and environmental mitigation strategies for acne vulgaris in Bangladesh.

## 1. Introduction

Skin diseases affect people of all ages, from newborns to the elderly. These conditions can harm individuals in various ways and may have a significant impact on both individuals and communities. Moreover, the distribution of skin diseases differs by countries and even within the same countries, there are regional differences [1]. Bangladesh, in particular, is a country with a lot of diversity in terms of climate, social position, religion, and culture [2]. While Bangladesh has achieved tremendous improvement in recent years in terms of overall health, with most health indices improving, the country’s skin health has not seen the same progress.

Globally, the overall point prevalence of any type of skin disease was 61.2% [3]. Zooming out to a broader regional perspective, South Asia, particularly India, had the highest number of new cases and deaths due to skin and subcutaneous diseases [1]. In 2019, South Asia recorded approximately 1.21 billion new cases of skin and subcutaneous diseases [4]. Indeed, the presence of skin diseases is among the most prevalent public health challenges in developing countries, and despite skin diseases having a high morbidity rate, they appear to have a low death rate [5]. Troublingly, half of the world lacks access to essential health services and hence lacks access to basic skin care [6]. Circling back to Bangladesh, the country is well‐known for its high rate of skin diseases [4]. In Bangladesh, due to a shortage of qualified and specialized doctors and practitioners, rural populations, particularly those in remote coastal areas, lack access to adequate healthcare, including dermatological treatment. While telemedicine holds promise as a means of bridging this gap in underserved villages, its current reach in Bangladesh remains far from satisfactory, leaving much of the rural population still without adequate healthcare access [7].

Over the years, skin diseases have piqued people’s curiosity throughout the world since they are common yet possibly avoidable and controlled. Although skin diseases are not as fatal as some of the more severe diseases, their contribution to total morbidity is significant, placing them as the fourth leading cause of nonfatal disease burden [8]. In spite of substantial regional variation, skin diseases are prevalent on a global scale. The overall incidence of skin diseases was around 7% [9]. However, other population‐based surveys found far higher prevalence rates; for example, 27% of European countries were afflicted with this condition [10]. Shockingly, it is estimated that between 21% and 87% of African children suffer from some form of skin disease, with dermatology and other medical specialists seeing as much as a third of all outpatients [11]. Delving deeper into the European data, the most prevalent skin diseases are fungal skin disease, followed by eczema, acne, alopecia, and psoriasis. Furthermore, alopecia, acne, eczema, and rosacea are more common among women, while psoriasis and sexually transmitted infections (STIs) are more common among men [12]. Meanwhile, in sub‐Saharan Africa, dermatophytosis, scabies, and bacterial infections are the most common skin diseases, whereas in South Asia, dermatophytosis, vitiligo, leprosy, and psoriasis predominate [4]. In Bangladesh, a community‐based study reported a skin disease prevalence of 15% among the general population [13]. The prevalent and persistent skin diseases among individuals from Bangladesh encompass fungal infections, psoriasis, atopic dermatitis, seborrheic dermatitis, and acne vulgaris [14].

Given the high prevalence and regional variations of skin diseases, detecting and managing these conditions is critical not only for treating patients but also for limiting the spread of infectious diseases. Consequently, several articles have focused on the management of skin diseases. To minimize the burden of skin diseases and enhance people’s quality of life, improvements in environmental sanitation, as well as public education on personal cleanliness and healthy living, are required [15]. Additionally, telemedicine has the potential to revolutionize the delivery of equitable dermatological treatments to disadvantaged communities. Considering this, a study had proposed low‐cost, store‐and‐forward tele‐dermatology to give skin care to hard‐to‐reach individuals, with the goal of improving skin health in Bangladesh [16].

To better understand and address the high prevalence of skin diseases, particularly in regions like Bangladesh, it is crucial to examine the factors that contribute to their incidence. Specifically, the incidence of skin diseases is influenced by lifestyle factors, food items, and environmental factors [17–20]. Furthermore, several studies have found a significant relationship between skin diseases and heritability [21, 22]. When examining lifestyle factors in detail, key determinants include living near poor drainage systems, contact with infected individuals, wearing unclean clothes, psychological issues, poor hygiene habits, weakened immune systems, use of illicit drugs or alcohol, insect bites, extended family, and use of cosmetics [23–33]. In terms of dietary influences, food items identified as potentially influencing skin health include specific types of fish (hilsa and shrimp), vegetables (brinjal, spinach, and arum), grains (lentil and corn), meats (beef, mutton, and duck), fruits (pineapple), and poultry products (chicken’s and duck’s eggs) [34–42]. Additionally, environmental factors such as congested living conditions, indoor air pollution, sun exposure, extremely heated weather, dust mites, water and air pollution, climate change, and humidity also play crucial roles [43–50].

When examining how these factors manifest in specific conditions, research shows that atopic dermatitis is significantly influenced by a combination of lifestyle factors, environmental factors, food items, and heritable factors [51–54]. In the case of fungal infections, both lifestyle and heritable factors play predominant roles in their development [55, 56]. Similarly, acne vulgaris shows strong associations with lifestyle, environmental, and heritable factors [57–59]. When examining seborrheic dermatitis, studies indicate that food items and heritable factors are significant contributing factors [60, 61]. Moreover, psoriasis demonstrates significant associations with lifestyle, environmental, and heritable factors, highlighting the complex interplay of multiple determinants in skin disease manifestation [62–64].

Beyond these general categorizations, demographic and personal factors play crucial roles in disease manifestation. Most notably, age emerges as one of the most influential factors across multiple skin conditions, playing a significant role in acne vulgaris, atopic dermatitis, and fungal infections [65–67]. Alongside age, gender, and sex considerations also show notable importance, specifically affecting both atopic dermatitis and fungal infections [65, 66]. From a demographic perspective, race and ethnicity demonstrate significant influence on both fungal infections and psoriasis [65, 68]. Expanding on socioeconomic factors, educational background appears to be a determining factor for both atopic dermatitis and psoriasis [66, 68]. On the genetic front, heritability shows strong associations with both acne vulgaris and atopic dermatitis [66, 67]. Considering environmental influences, living conditions play a specific role in atopic dermatitis, with both residence type and house type being significant factors [66, 69, 70]. In the healthcare domain, herbal medication usage is linked to atopic dermatitis, while health insurance status shows a significant relationship with psoriasis [66, 68]. With behavioral factors, lifestyle choices cluster exclusively around seborrheic dermatitis, along with smoking, alcohol consumption, and stress levels all showing significant associations with this condition, while socioeconomic status emerges independently as a significant factor specifically for fungal infections [65, 71].

This complex interplay of factors underscores the multifaceted nature of skin disease etiology. However, to the best of our knowledge, hardly any study was found to detect the lifestyle, environmental, food items, and heritable factors that are related to certain skin diseases in Bangladesh. Therefore, to address the challenges mentioned earlier and improve skin health in the country, it is necessary to conduct a large‐scale study on this topic in Bangladesh. With this in mind, we set up our main objective to identify the lifestyle, environmental, food items, and heritable factors that are associated with certain skin diseases in Bangladesh.

## 2. Methodology

### 2.1. Sampling Technique

This cross‐sectional study employed a purposive quota sampling strategy across three divisions of Bangladesh: Dhaka, Rangpur, and Chattogram. This methodology was selected for its effectiveness in recruiting participants from specific geographic subgroups who could provide meaningful insights relevant to the research objectives [72]. The selection of these three divisions ensured geographic diversity, representing central, northern, and southeastern regions of Bangladesh.

To facilitate robust division‐level statistical comparisons, an equal allocation approach was implemented, targeting approximately 200 participants from each division. This strategy was adopted to ensure sufficient statistical power for examining geographic variations in skin disease patterns and risk factors. Given the absence of comprehensive administrative data on the prevalence of factor‐induced skin diseases across Bangladesh’s divisions, quota allocation was determined through consultation among the research team, considering both feasibility and the need for adequate representation across regions.

### 2.2. Sample Size Determination

Sample size was calculated using the standard formula for single proportion estimation [73].

(1) n=Z2×p×1−pd2.

Parameters: *Z* = 1.96 (95% confidence level); *p* = 0.15 (estimated skin disease prevalence based on previous literature) [13]; *d* = 0.025 (margin of error).

Calculation:

(2) n=1.962×0.15×10.15−0.0252≈784.

The study enrolled 788 participants (Dhaka: *n* = 263; Rangpur: *n* = 249; Chattogram: *n* = 276), meeting the minimum required sample size for the specified precision.

### 2.3. Data Collection

A cross‐sectional study was conducted across the three divisions between December 2022 and July 2023 using a structured questionnaire administered through face‐to‐face interviews. The comprehensive survey tool was designed to gather information on skin disease status, demographic and basic characteristics, lifestyle factors, food items, environmental factors, and heritable factors.

### 2.4. Interviewer Training and Data Collection Procedure

Data were collected by the research team members who received comprehensive training prior to fieldwork. The training included:

• Familiarization with the questionnaire structure and content.
• Techniques for obtaining informed consent.
• Standardized procedures for administering questions to ensure consistency across all data collectors.
• Protocols for handling sensitive questions.
• Data recording procedures.

Mock interviews were conducted during training sessions to ensure all research team members understood proper administration techniques and could address common participant queries. This standardized training ensured consistency in data collection across all three divisions.

### 2.5. Data Quality Control

Several measures were implemented to ensure data quality throughout the study. The principal investigator conducted daily supervision and review of completed questionnaires for completeness and consistency. Questionnaires with missing or inconsistent responses were flagged and clarified with participants when possible or excluded from analysis if clarification was not feasible. Double data entry was performed by two independent data entry operators, and discrepancies were resolved through verification against the original questionnaires. Range checks and logical consistency checks were performed during data cleaning to identify and correct errors. Regular team meetings were held throughout the data collection period to address any challenges and ensure adherence to standardized protocols.

### 2.6. Instrument Development and Validation

The structured questionnaire was initially developed in English based on existing literature and expert consultation with public health professionals. The questionnaire was then translated into Bengali (Bangla), the native language of Bangladesh, to ensure participant comprehension. The translation process involved:

• Forward translation from English to Bengali by a bilingual public health professional.
• Review of the Bengali version by the research team to ensure cultural appropriateness and clarity.
• Back‐translation to English by an independent translator to verify accuracy.

Discrepancies between the original and back‐translated versions were discussed and resolved. The final Bengali questionnaire was used for all data collection to ensure participants fully understood the questions. No formal pilot study was conducted prior to data collection. Face validity was established through review by public health expert to ensure the questionnaire adequately covered relevant risk factors for skin diseases in the Bangladeshi context.

Participants were first asked whether they currently had any skin disease (Yes/No). Those responding ‘Yes’ were then asked to identify their specific condition(s) from a list: acne vulgaris, atopic dermatitis, fungal infections, seborrheic dermatitis, and psoriasis. In developing countries, research often relies on self‐reported data due to various constraints, including resource scarcity, cultural and social barriers, political and economic influences, infrastructure deficiencies, human resource challenges, and operational obstacles [74]. We acknowledge that this self‐identification approach, without clinical verification or symptom‐based screening, may have resulted in substantial misclassification. Participants may have had difficulty understanding medical terminology or distinguishing between similar conditions. However, due to the lack of dermatological specialists in rural areas and these resource constraints, clinical confirmation was not feasible for this large‐scale community survey.

Binary outcome variables (Yes = 1, No = 0) were created for overall skin disease status and each specific condition, with participants able to report multiple conditions simultaneously.

### 2.7. Ethical Approval and Consent

This study was approved by the SUST Research Center Ethics Committee of Shahjalal University of Science and Technology, Sylhet, Bangladesh (Ethics Approval No.: PS/2022/2/34). All participants provided verbal informed consent prior to data collection. The researchers explained the study’s purpose, voluntary nature of participation, and confidentiality measures before administering the questionnaire. Verbal consent was obtained due to varying literacy levels among the study population and is standard practice for community‐based surveys in Bangladesh. All data were anonymized during entry to protect participant confidentiality. The informed consent statement and questionnaire are provided in the Supporting Information (available here).

### 2.8. Statistical Analyses

The analyses of the surveyed data include univariate tools (frequency, percentage, and chi‐square test) and multivariate tools (principal component analysis [PCA] and principal component logistic regression [PCLR]). The combination of these analytical methods allows for both broad overviews and in‐depth insights into the factors associated with certain skin diseases in the studied population.

PCA was performed using Pearson correlation matrices calculated from the binary indicator variables. PCA combined multiple related risk factors within each domain (lifestyle, environmental, food, and heritable) into a single overall measure. For example, instead of examining family history in father, mother, siblings, and grandparents separately, these were combined into a single heritable factor measure, with higher values indicating more family members with skin disease. We acknowledge that PCA was originally developed for continuous data and that polychoric correlations represent a theoretically superior alternative for binary inputs [75]. However, PCA on binary indicators has been widely applied in health and epidemiological research and remains an accepted practical approach when the goal is index construction rather than precise variance decomposition [76, 77]. In our data, tetrachoric correlations, theoretically designed for binary variables, yielded unacceptable Kaiser–Meyer–Olkin (KMO) values (< 0.60), indicating poor suitability for factor extraction, while Pearson correlations yielded acceptable diagnostic statistics. The suitability of PCA for each factor category was assessed using the KMO measure of sampling adequacy and Bartlett’s test of sphericity. KMO values were 0.75 for lifestyle factors (middling), 0.61 for environmental factors (mediocre but acceptable), 0.61 for food items (mediocre but acceptable), and 0.61 for heritable factors (mediocre but acceptable), all exceeding the acceptable threshold of 0.60. Bartlett’s test of sphericity was highly significant for all factor categories (*χ* ^2^ = 1926.06, df = 66, *p* < 0.001 for lifestyle; *χ* ^2^ = 1006.70, df = 55, *p* < 0.001 for environmental; *χ* ^2^ = 1192.17, df = 78, *p* < 0.001 for food items; *χ* ^2^ = 692.11, df = 78, *p* < 0.001 for heritable factors), confirming that the Pearson correlation matrices were suitable for factor extraction in this dataset. The derived first PC_1_ from each domain was subsequently used in PCLR models, wherein all four factor domains were tested independently against each skin disease outcome in both unadjusted and confounder‐adjusted models. Factor‐disease combinations were retained for final interpretation only when adequate model fit was demonstrated by both the omnibus likelihood ratio test and the Hosmer–Lemeshow goodness‐of‐fit test.

For descriptive analyses, the overall prevalence of skin disease was calculated as the proportion of all respondents reporting any skin condition (*n* = 788), while the prevalence of specific skin diseases (atopic dermatitis, fungal infection, acne vulgaris, seborrheic dermatitis, and psoriasis) was calculated as proportions among those who reported having any skin disease (*n* = 487).

To assess potential confounding by sociodemographic factors, we conducted sensitivity analyses by fitting multivariable logistic regression models. These models adjusted for variables that showed significant bivariate associations with each outcome in Table 1, plus core demographics (age group, sex, and division) for all models. Specifically, models for fungal infection adjusted for profession and house type; models for atopic dermatitis adjusted for house type; models for acne vulgaris adjusted for education and profession; models for seborrheic dermatitis adjusted for marital status, education, profession, and water use; and models for psoriasis adjusted for marital status, education, profession, and house type.

**TABLE 1:** Sociodemographic characteristics of the respondents.

Characteristics		Responses			Chi‐square	df	*p* value
		No *N* (%)	Yes *N* (%)	Total *N* (%)			
*Atopic dermatitis*							
Type of the house	Flat	62 (45.59%)	128 (36.47%)	252 (31.98%)	11.41	3	0.01
	Building	44 (32.35%)	108 (30.77%)	258 (32.74%)			
	Half‐building	24 (17.65%)	62 (17.66%)	155 (19.67%)			
	Tin/Slum	6 (4.41%)	53 (15.10%)	123 (15.61%)			

*Fungal infection*							
Occupation	Job	55 (15.45%)	24 (18.32%)	125 (15.86%)	25.33	6	< 0.01
	Business	26 (7.30%)	26 (19.85%)	74 (9.39%)			
	Farming	11 (3.09%)	1 (0.76%)	16 (2.03%)			
	Housewife	113 (31.74%)	31 (23.66%)	215 (27.28%)			
	Student	140 (39.33%)	43 (32.82%)	291 (36.93%)			
	Auto driver	0 (0%)	2 (1.53%)	13 (1.65%)			
	Others	11 (3.09%)	4 (3.05%)	54 (6.85%)			

Type of the house	Flat	151 (42.42%)	39 (29.77%)	252 (31.98%)	11.53	3	0.01
	Building	108 (30.34%)	44 (33.59%)	258 (32.74%)			
	Half‐building	52 (14.61%)	34 (25.95%)	155 (19.67%)			
	Tin/Slum	45 (12.64%)	14 (10.69%)	123 (15.61%)			

*Acne vulgaris*							
Age (in years)	< 18	34 (8.81%)	7 (6.93%)	90 (11.42%)	12.39	3	0.01
	18–30	164 (42.49%)	59 (58.42%)	356 (45.18%)			
	31–40	64 (16.58%)	19 (18.81%)	131 (16.62%)			
	> 40	124 (32.12%)	16 (15.84%)	211 (26.78%)			

Education	Below SSC	144 (37.31%)	23 (22.77%)	311 (39.47%)	12.81	4	0.01
	SSC‐HSC	84 (21.76%)	17 (16.83%)	157 (19.92%)			
	B.Sc.	109 (28.24%)	43 (42.57%)	218 (27.66%)			
	M.Sc.	39 (10.10%)	15 (14.85%)	81 (10.28%)			
	Higher Study	10 (2.59%)	3 (2.97%)	21 (2.66%)			

Occupation	Job	58 (15.03%)	21 (20.79%)	125 (15.86%)	20.78	6	< 0.01
	Business	45 (11.66%)	7 (6.93%)	74 (9.39%)			
	Farming	12 (3.11%)	0 (0%)	16 (2.03%)			
	Housewife	123 (31.87%)	21 (20.79%)	215 (27.28%)			
	Student	131 (33.94%)	52 (51.49%)	291 (36.93%)			
	Auto driver	2 (0.52%)	0 (0%)	13 (1.65%)			
	Others	15 (3.89%)	0 (0%)	54 (6.85%)			

*Seborrheic dermatitis*							
Marital status	Unmarried	188 (44.66%)	19 (28.79%)	340 (43.15%)	5.25	1	0.02
	Married	233 (55.34%)	47 (71.21%)	448 (56.85%)			

Education	Below SSC	146 (34.68%)	21 (31.82%)	311 (39.47%)	17.82	4	< 0.01
	SSC‐HSC	88 (20.90%)	13 (19.70%)	157 (19.92%)			
	B.Sc.	138 (32.78%)	14 (21.21%)	218 (27.66%)			
	M.Sc.	37 (8.79%)	17 (25.76%)	81 (10.28%)			
	Higher Study	12 (2.85%)	1 (1.52%)	21 (2.66%)			

Occupation	Job	63 (14.96%)	16 (24.24%)	125 (15.86%)	15.80	6	0.02
	Business	38 (9.03%)	14 (21.21%)	74 (9.39%)			
	Farming	10 (2.38%)	2 (3.03%)	16 (2.03%)			
	Housewife	132 (31.35%)	12 (18.18%)	215 (27.28%)			
	Student	163 (38.72%)	20 (30.30%)	291 (36.93%)			
	Auto driver	2 (0.48%)	0 (0%)	13 (1.65%)			
	Others	13 (3.09%)	2 (3.03%)	54 (6.85%)			

Source of usable water	Tubewell	133 (31.59%)	27 (40.91%)	285 (36.17%)	6.94	2	0.03
	Supply	258 (61.28%)	30 (45.45%)	390 (49.49%)			
	Submersible pump	30 (7.13%)	9 (13.64%)	113 (14.34%)			

*Psoriasis*							
Age (in years)	< 18	41 (9.17%)	0 (0%)	90 (11.42%)	14.88	3	< 0.01
	18–30	212 (47.43%)	11 (27.50%)	356 (45.18%)			
	31–40	70 (15.66%)	13 (32.50%)	131 (16.62%)			
	> 40	124 (27.74%)	16 (40%)	211 (26.78%)			

Marital status	Unmarried	199 (44.52%)	8 (20%)	340 (43.15%)	8.06	1	< 0.01
	Married	248 (55.48%)	32 (80%)	448 (56.85%)			

Education	Below SSC	162 (36.24%)	5 (12.50%)	311 (39.47%)	58.67	4	< 0.01
	SSC‐HSC	74 (16.55%)	27 (67.50%)	157 (19.92%)			
	B.Sc.	145 (32.44%)	7 (17.50%)	218 (27.66%)			
	M.Sc.	53 (11.86%)	1 (2.50%)	81 (10.28%)			
	Higher Study	13 (2.91%)	0 (0%)	21 (2.66%)			

Occupation	Job	79 (17.67%)	0 (0%)	125 (15.86%)	34.64	6	< 0.01
	Business	40 (8.95%)	12 (30%)	74 (9.39%)			
	Farming	12 (2.68%)	0 (0%)	16 (2.03%)			
	Housewife	124 (27.74%)	20 (50%)	215 (27.28%)			
	Student	175 (39.15%)	8 (20%)	291 (36.93%)			
	Auto driver	2 (0.45%)	0 (0%)	13 (1.65%)			
	Others	15 (3.36%)	0 (0%)	54 (6.85%)			

Type of the house	Flat	168 (37.58%)	22 (55%)	252 (31.98%)	10.87	3	0.01
	Building	140 (31.32%)	12 (30%)	258 (32.74%)			
	Half‐building	86 (19.24%)	0 (0%)	155 (19.67%)			
	Tin/Slum	53 (11.86%)	6 (15%)	123 (15.61%)			

*Note: N* = frequency.

Abbreviation: df, degrees of freedom.

These analyses were conducted using R Version 4.3.2. Specific packages used included psych for PCA; stats (base R) for logistic regression modeling; ResourceSelection for Hosmer–Lemeshow goodness‐of‐fit testing; lmtest for likelihood ratio tests; dplyr for data manipulation; ggplot2 and ggtext for data visualization; and haven for importing SPSS data files.

## 3. Results

**FIGURE 1:**
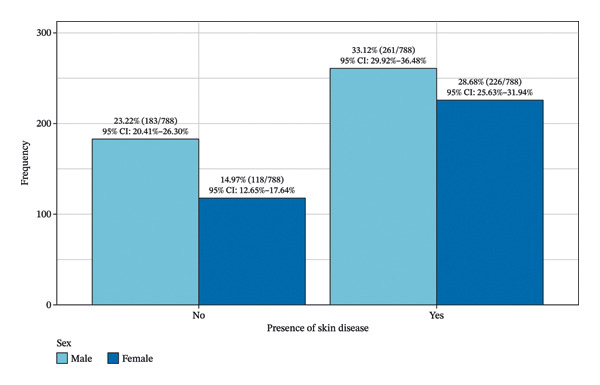
Presence of skin diseases by gender.

As shown in Figure 1, 61.80% of all respondents reported skin disease during the survey period, with males accounting for 33.12% and females for 28.68% of the total sample. The remaining 38.20% reported no skin disease, consisting of 23.22% males and 14.97% females.

**FIGURE 2:**
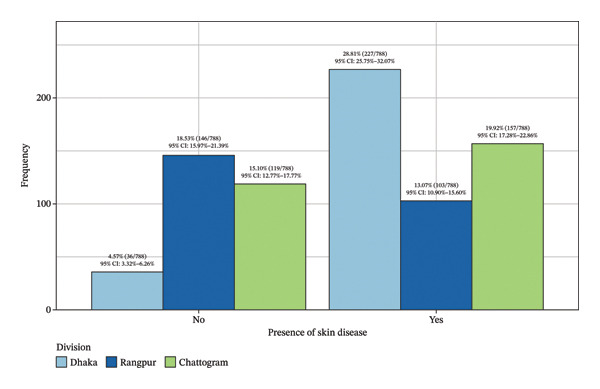
Presence of skin diseases by division.

Furthermore, Figure 2 depicts the distribution of respondents with skin disease categorized by division. Notably, most of the positive cases were concentrated in Dhaka, which accounted for 28.81% of all respondents, followed by Chattogram and Rangpur, which accounted for 19.92% and 13.07% of all respondents, respectively.

**FIGURE 3:**
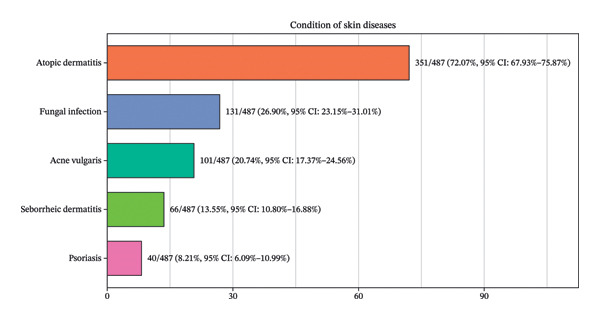
Status of specific skin diseases (percentages do not sum to 100% as respondents could report multiple concurrent skin conditions).

Figure 3 shows that among respondents who reported having any skin disease (*n* = 487), atopic dermatitis was the most prevalent condition, affecting 72.07% of these individuals, followed by fungal infection (26.90%), acne vulgaris (20.74%), seborrheic dermatitis (13.55%), and psoriasis (8.21%). As respondents could report multiple concurrent conditions, these percentages are not mutually exclusive and do not sum to 100%.

Table 1 presents the sociodemographic distribution of different skin diseases. The analysis includes various sociodemographic factors such as age groups, marital status, education level, profession, source of usable water, and type of house. The table specifically illustrates only the statistically significant associations (*p* < 0.05 at 5% level) between these sociodemographic factors and certain skin diseases.

Atopic dermatitis presents a significant public health concern, as evidenced by its educational demographics, with 34.47% of patients having below SSC education, while 33.62% hold B.Sc. degrees. In terms of living conditions, housing data reveals that 36.47% of these patients reside in flats, 30.77% in buildings, 17.66% in half‐buildings, and 15.10% in tin/slum dwellings.

When examining fungal infections and their occupational distribution, students make up the largest group at 32.82%, followed by housewives at 23.66%, business professionals at 19.85%, and job holders at 18.32%. Regarding living conditions, the housing distribution for fungal infections shows that 33.59% live in buildings, 29.77% in flats, 25.95% in half‐buildings, and 10.69% in tin/slum structures.

Shifting focus to acne vulgaris, the age demographics show this condition is most prevalent among individuals aged 18–30 years (58.42%), followed by those aged 31–40 years (18.81%), over 40 years (15.84%), and under 18 years (6.93%). In terms of educational background, a significant portion of patients (42.57%) holds B.Sc. degrees, while 22.77% are below SSC, 16.83% have SSC‐HSC qualifications, and 14.85% possess M.Sc. degrees. Regarding occupational distribution, students represent the largest professional group at 51.49%, with jobholders and housewives each comprising 20.79%, and business professionals at 6.93%.

Moving on to seborrheic dermatitis, analysis of marital status reveals that a significant majority (71.21%) are married while 28.79% remain unmarried. With respect to educational attainment, the distribution shows that 31.82% have below SSC qualifications, followed by those with M.Sc. (25.76%), B.Sc. (21.21%), and SSC‐HSC (19.70%). In terms of occupation, students account for 30.30%, job holders for 24.24%, business professionals for 21.21%, and housewives for 18.18%.

Finally, examining psoriasis cases, age distribution reveals that 40% of patients are over the age of 40 years, while those aged between 31–40 years constitute 32.50%, and those aged between 18–30 years make up the remaining 27.50%. Regarding marital status, a significant majority (80%) are married. In terms of education, most patients (67.50%) have SSC‐HSC qualifications, with B.Sc holders at 17.50% and those below SSC at 12.50%. Looking at occupational patterns, housewives represent half of the cases (50%), followed by business professionals at 30%, and students at 20%. The housing distribution reveals that most live in flats (55%), followed by buildings (30%) and tin/slum structures (15%).

We conducted a multiple response analysis, and Table 2 summarizes the results, showing various factors indicated by the respondents that might trigger their skin diseases. Notably, since one respondent might refer to multiple responses for the occurrence of skin diseases, the total responses increased from 788 to 3090 for lifestyle factors, 3597 for environmental factors, 806 for food items, and 1007 for heritable factors. Regarding lifestyle factors, remarkably, living near a poor drainage system emerged as the most frequently cited (54.05%), closely trailed by contact with infected individuals (53.92%). Other notable factors included wearing unclean clothes (49.22%), anxiety or depression (44.91%), sudden anger (44.52%), showering too little (40.99%), and excessive sweating (38.25%). In terms of environmental factors, exposure to sunlight in the room emerged as the most frequently cited factor (81.56%), closely followed by extremely heated weather (78.62%). Other notable environmental triggers included dust mites (56.08%), polluted water (48.66%), climate change (44.05%), and polluted air (39.18%). Concerning food items, beef emerged as the most frequently cited factor (65.36%), closely trailed by hilsa (48.60%), brinjal (45.53%), and shrimp (20.95%). Lastly, among heritable factors, having a sister with skin diseases emerged as the most frequently cited factor (39.59%), closely followed by mother (39.02%), father (33.58%), and brother (24.77%).

**TABLE 2:** Multiple response analysis of various factors.

Variables	Responses		Percent of cases (%)
	*N*	Percent (%)	
*Lifestyle factors*			
Anxiety or depression	344	11.13	44.91
Sudden anger	341	11.04	44.52
Showering too little	314	10.16	40.99
Excessive sweating	293	9.48	38.25
Wearing unclean clothes	377	12.20	49.22
Weakened immune system	197	6.38	25.72
Use illicit drugs or alcohol	123	3.98	16.06
Contact with infected individuals	413	13.37	53.92
Affected by insect bites	117	3.79	15.27
Use cosmetics	88	2.85	11.49
Larger family size (extended family)	69	2.23	9.01
Living near poor drainage system	414	13.40	54.05
Total	3090	100.00	403.39

*Environmental factors*			
Living in an area with lack of air‐conditioning	180	5.00	23.05
Living in a room under thermal discomfort	163	4.53	20.87
Exposure to heavy metal in indoor air	59	1.64	7.55
Exposure to sunlight in the room	637	17.71	81.56
Dust mites	438	12.18	56.08
Extremely heated weather	614	17.07	78.62
Cold weather	241	6.70	30.86
Humidity	235	6.53	30.09
Polluted air	306	8.51	39.18
Polluted water	380	10.56	48.66
Climate change	344	9.56	44.05
Total	3597	100.00	460.56

*Food items*			
Hilsa	174	21.59	48.60
Shrimp	75	9.31	20.95
Brinjal	163	20.22	45.53
Spinach	16	1.99	4.47
Arum	19	2.36	5.31
Lentil	12	1.49	3.35
Corn	20	2.48	5.59
Beef	234	29.03	65.36
Mutton	29	3.60	8.10
Duck	15	1.86	4.19
Pineapple	15	1.86	4.19
Chicken’s egg	16	1.99	4.47
Duck’s egg	18	2.23	5.03
Total	806	100.00	225.14

*Heritable factors*			
Father	179	17.78	33.58
Mother	208	20.66	39.02
Brother	132	13.11	24.77
Sister	211	20.95	39.59
Grandfather	28	2.78	5.25
Grandmother	43	4.27	8.07
Paternal cousin	48	4.77	9.01
Maternal cousin	23	2.28	4.32
Husband	22	2.18	4.13
Wife	26	2.58	4.88
Son	21	2.09	3.94
Daughter	21	2.09	3.94
None	45	4.47	8.44
Total	1007	100.00	188.93

*Note: N* = frequency.

To further investigate the underlying factors related to certain skin diseases of the respondents, we performed PCA, as shown in Table 3. It illustrates the correlations between the original data and each PC. In our analysis, we focused on the most significant relationships by selecting the correlation of the original variables/factors with a threshold of |0.30| or greater, suppressing values smaller than this threshold.

**TABLE 3:** Loadings (suppressed <|0.30|) of principal component analysis.

	**PC_1_**	**PC_2_**	**PC_4_**	**PC_3_**	**PC_6_**	**PC_5_**

*Lifestyle factors*						
Anxiety or depression				0.83		
Sudden anger			0.72			0.30
Showering too little		0.82				
Excessive sweating			0.82			
Wearing unclean clothes		0.86				
Weakened immune system					0.88	
Use illicit drugs or alcohol	0.82					
Contact with infected individuals		0.34		0.58	0.47	
Affected by insect bites	0.80					
Use cosmetics	0.82					
Larger family size (extended family)						0.94
Living near poor drainage system		−0.52		0.34		
Proportion variance	0.18	0.17	0.11	0.10	0.10	0.09
Cumulative variance	0.18	0.35	0.46	0.56	0.65	0.74

	**PC**_**1**_	**PC**_**3**_	**PC**_**2**_	**PC**_**5**_	**PC**_**4**_	**PC**_**6**_

*Environmental factors*						
Living in an area with lack of air‐conditioning	0.81					
Living in a room under thermal discomfort	0.86					
Exposure to heavy metal in indoor air						0.91
Exposure to sunlight in the room					0.86	
Dust Mites		0.57			0.46	
Extremely heated weather			0.82			
Cold weather				0.93		
Humidity			0.59	0.38		0.30
Polluted air		0.85				
Polluted water		0.71				
Climate change	−0.33		0.51	0.32	−0.38	
Proportion variance	0.15	0.15	0.13	0.11	0.11	0.09
Cumulative variance	0.15	0.30	0.43	0.53	0.64	0.73

	**PC**_**1**_	**PC**_**2**_	**PC**_**4**_	**PC**_**3**_	**PC**_**5**_	**PC**_**6**_

*Food items*						
Hilsa	0.81					
Shrimp			0.54	0.45		
Brinjal	0.80					
Spinach				0.70		
Arum			0.60			
Lentil				0.63		
Corn						0.95
Beef	0.79					
Mutton					0.74	
Duck		0.86				
Pineapple			0.73			
Chicken’s egg					0.76	
Duck’s egg		0.86				
Proportion variance	0.15	0.12	0.11	0.09	0.09	0.08
Cumulative variance	0.15	0.27	0.38	0.48	0.57	0.65

	**PC**_**1**_	**PC**_**4**_	**PC**_**2**_	**PC**_**3**_	**PC**_**6**_	**PC**_**5**_

*Heritable factors*						
Father	0.74					
Mother	0.78					
Brother	0.32	0.65				
Sister	0.48	0.51				
Grandfather			0.84			
Grandmother			0.83			
Paternal cousin		0.82				
Maternal cousin					0.94	
Husband						0.88
Wife				0.46		
Son				0.74		
Daughter				0.69		
None	−0.33					−0.48
Proportion variance	0.13	0.11	0.11	0.10	0.08	0.08
Cumulative variance	0.13	0.24	0.35	0.45	0.53	0.62

Abbreviation: PC, principal component.

The PCA revealed distinct patterns across different factor categories. For lifestyle factors, six PCs collectively accounted for 74% of data variation, with PC_1_, PC_2_, PC_4_, PC_3_, PC_6_, and PC_5_ individually accounting for 18%, 17%, 11%, 10%, 10%, and 9% of data variation, respectively. Similarly, for environmental factors, six PCs collectively explained 73% of data variation. In this case, PC_1_, PC_3_, PC_2_, PC_5_, PC_4_, and PC_6_ individually accounted for 15%, 15%, 13%, 11%, 11%, and 9% of data variation, respectively. Moving on to food items, the analysis showed that six PCs collectively accounted for 65% of data variation, with PC_1_, PC_2_, PC_4_, PC_3_, PC_5_, and PC_6_ individually explaining 15%, 12%, 11%, 9%, 9%, and 8% of data variation, respectively. Lastly, for heritable factors, six PCs collectively accounted for 62% of data variation. In this category, PC_1_, PC_4_, PC_2_, PC_3_, PC_6_, and PC_5_ individually accounted for 13%, 11%, 11%, 10%, 8%, and 8% of data variation, respectively. Notably, compared to continuous variables, categorical variables exhibit more pronounced variation [75].

As PCs are continuous variables, this variability is reflected in Table 3. According to Table 3, the first PC_1_ consistently accounted for the highest variation in the data across all factor categories. Consequently, PC_1_ emerged as the most vital among the six PCs identified. For lifestyle factors, PC_1_ correlates with three original factors. Specifically, the values of PC_1_ for factors: affected by insect bites, use illicit drugs or alcohol, and use cosmetics were found to be 0.80, 0.82, and 0.82, respectively. Similarly, PC_1_ for environmental factors correlates with three original factors. The values of PC_1_ rise with the factors: climate change, living in an area with a lack of air‐conditioning, and living in a room under thermal discomfort, as they were found to be −0.33, 0.81, and 0.86, respectively. Regarding food items, PC_1_ correlates with three original factors. The values of PC_1_ rise with the factors: beef, brinjal, and hilsa, as they were found to be 0.79, 0.80, and 0.81, respectively. Lastly, for heritable factors, PC_1_ correlates with four original factors. The values of PC_1_ rise with the factors: brother, sister, father, and mother, as they were found to be 0.32, 0.48, 0.74, and 0.78, respectively.

We further employed PCLR to explore the contribution of underlying patterns in each group of variables (lifestyle factors, environmental factors, food items, and heritable factors) to the development of each skin disease outcome. PCA was used to reduce the dimensionality of variables within each domain into a single first PC_1_, which was subsequently used as a continuous covariate in logistic regression models with skin disease status as the binary outcome (Yes = 1, No = 0). For each skin disease, all four factor domains were tested independently in both unadjusted models (PC_1_ only) and adjusted models (PC_1_ and disease‐specific confounders identified from preliminary analyses). Confounders adjusted for each disease were selected based on statistically significant associations identified in descriptive analyses. The omnibus likelihood ratio test and Hosmer–Lemeshow goodness‐of‐fit test were used to evaluate model fit, and only factor‐disease combinations demonstrating adequate fit in both unadjusted and adjusted models were retained for final interpretation.

Table 4 presents data on model fitting, focusing on the statistical significance of chi‐square values for the fitted models. The analysis includes lifestyle factors, environmental factors, food items, and heritable factors associated with certain skin diseases. The table specifically illustrates only the statistically significant associations (*p* < 0.05 at 5% level) between these factors and different skin diseases.

**TABLE 4:** Goodness of fit tests.

Skin disease	Factor	Chi‐square	df	Test	Value
*Unadjusted*					
Fungal infection	Heritable factors	7.30	1	Omnibus Test	0.01
				Hosmer and Lemeshow Test	0.13

Atopic dermatitis	Food factors	29.96	1	Omnibus Test	< 0.01
				Hosmer and Lemeshow Test	0.01

Acne vulgaris	Lifestyle factors	13.17	1	Omnibus Test	< 0.01
				Hosmer and Lemeshow Test	0.61
	Environmental factors	10.90	1	Omnibus Test	< 0.01
				Hosmer and Lemeshow Test	0.05

Seborrheic dermatitis	Heritable factors	7.48	1	Omnibus Test	0.01
				Hosmer and Lemeshow Test	0.39

Psoriasis	Lifestyle factors	14.26	1	Omnibus Test	< 0.01
				Hosmer and Lemeshow Test	< 0.01
	Environmental factors	29.35	1	Omnibus Test	< 0.01
				Hosmer and Lemeshow Test	< 0.01
	Heritable factors	21.23	1	Omnibus Test	< 0.01
				Hosmer and Lemeshow Test	< 0.01

*Adjusted*					
Fungal infection	Heritable factors	7.17	1	Omnibus Test	0.01
				Hosmer and Lemeshow Test	0.27

Atopic dermatitis	Food factors	37.08	1	Omnibus Test	< 0.01
				Hosmer and Lemeshow Test	0.01

Acne vulgaris	Lifestyle factors	7.49	1	Omnibus Test	0.01
				Hosmer and Lemeshow Test	0.01

Acne vulgaris	Environmental factors	5.62	1	Omnibus Test	0.02
				Hosmer and Lemeshow Test	0.76

Seborrheic dermatitis	Heritable factors	5.40	1	Omnibus Test	0.02
				Hosmer and Lemeshow Test	0.04

Psoriasis	Lifestyle factors	9.89	1	Omnibus Test	< 0.01
				Hosmer and Lemeshow Test	0.01
	Environmental factors	20.37	1	Omnibus Test	< 0.01
				Hosmer and Lemeshow Test	< 0.01
	Food factors	8.94	1	Omnibus Test	< 0.01
				Hosmer and Lemeshow Test	< 0.01
	Heritable factors	16.91	1	Omnibus Test	< 0.01
				Hosmer and Lemeshow Test	0.01

Abbreviation: df, degrees of freedom.

The unadjusted fitted model for fungal infection shows significance for heritable factors in the Omnibus test (*χ* ^2^ = 7.30, df = 1, *p* = 0.01), suggesting a substantial impact of the model’s coefficients [78]. The Hosmer and Lemeshow test yielded a *p* value of 0.13 (> 0.05), indicating good model fit [79]. After adjusting for key sociodemographic confounders, the model retained significance in the Omnibus test (*χ* ^2^ = 7.17, df = 1, *p* = 0.01), and the Hosmer and Lemeshow test confirmed good calibration (*p* = 0.27, > 0.05), demonstrating that this association is robust to adjustment.

The unadjusted fitted model for acne vulgaris with environmental factors shows significance in the Omnibus test (*χ* ^2^ = 10.90, df = 1, *p* < 0.01) and marginal calibration in the Hosmer and Lemeshow test (*p* = 0.05). After adjustment, the model retained Omnibus significance (*χ* ^2^ = 5.62, df = 1, *p* = 0.02) and demonstrated good fit (*p* = 0.76, > 0.05), confirming this as a robust association.

**FIGURE 4:**
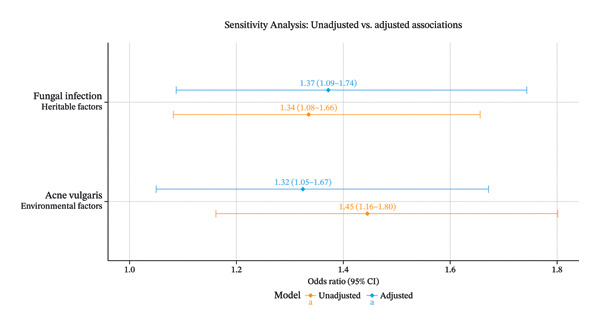
Sensitivity analysis: Unadjusted and adjusted odds ratios with 95% confidence intervals of the first principal components for associations with adequate model fit.

Accordingly, the two associations demonstrating good model fit in the adjusted models: fungal infection (heritable factors) and acne vulgaris (environmental factors), are carried forward into the sensitivity analysis presented in Figure 4, which displays the unadjusted and adjusted odds ratios side‐by‐side for these associations. The remaining associations, including atopic dermatitis (food factors), seborrheic dermatitis (heritable factors), acne vulgaris (lifestyle factors), and all psoriasis associations, were excluded due to poor model fit and/or loss of significance after adjustment.

Figure 4 presents both unadjusted and adjusted odds ratios with 95% confidence intervals of the first PCs for the two associations that demonstrated adequate goodness of fit in the adjusted models.

In the unadjusted analyses, heritable factors were significantly associated with fungal infection (OR: 1.34, 95% CI: 1.08–1.66), indicating that a one‐unit increase in PC_1_ representing heritable factors was associated with a 34% increase in the odds of fungal infection. Environmental factors were significantly associated with acne vulgaris (OR: 1.45, 95% CI: 1.16–1.80), suggesting a 45% increase in odds. After adjusting for sociodemographic confounders, both associations persisted and retained statistical significance. For fungal infection, the association with heritable factors remained significant after adjusting for age group, sex, division, profession, and house type (adjusted OR: 1.37, 95% CI: 1.09–1.74), with only marginal attenuation from the unadjusted estimate, confirming the robustness of this association. The association between acne vulgaris and environmental factors remained significant after adjustment (adjusted OR: 1.32, 95% CI: 1.05–1.67), suggesting that environmental influences on acne vulgaris are independent of sociodemographic factors. It is worth noting that atopic dermatitis (food factors), seborrheic dermatitis (heritable factors), acne vulgaris (lifestyle factors), and all psoriasis associations (lifestyle, environmental, food, and heritable factors) were excluded from the sensitivity analysis due to inadequate goodness of fit in the adjusted models and/or loss of statistical significance after adjustment, suggesting that those unadjusted associations were at least partially driven by sociodemographic confounding or inadequate model calibration rather than the PCs themselves.

## 4. Discussion

This study aims to identify factors associated with certain skin diseases in Bangladesh, revealing significant findings that contribute to the understanding of skin health in the region. Specifically, the results indicate that atopic dermatitis is the most prevalent skin disease (72.07%), followed by fungal infection (26.90%), acne vulgaris (20.74%), seborrheic dermatitis (13.55%), and psoriasis (8.21%). These findings align with previous research highlighting the predominance of certain skin diseases in South Asia [4]. Notably, this study finds an overall prevalence of skin diseases among respondents at 61.80%, considerably higher than previously reported 15% for Bangladesh [13].

In examining the various contributing factors, the research reveals significant associations between certain skin diseases and various sociodemographic factors, including age, education, marital status, house type, profession, and source of usable water. The analysis reveals house type as the significant sociodemographic factor for atopic dermatitis, a finding supported by multiple studies that identified these same factors as significant [31, 47, 66, 69, 70]. For fungal infections, occupation and house type emerge as key sociodemographic factors, aligning with previous research that highlighted occupation and house type as crucial determinants [31, 32, 47, 65]. The study identifies age, education, and occupation as primary factors for acne vulgaris, reinforcing existing literature that emphasized these same variables [31, 47, 67]. In the case of seborrheic dermatitis, the data points to marital status, education, occupation, and source of usable water as significant factors, consistent with prior studies that emphasized education, occupation, and water source [31, 32, 44, 45, 47, 71]. The analysis of psoriasis reveals age, marital status, education, occupation, and house type as key determinants, paralleling previous research findings [31–33, 45, 47, 68].

Beyond sociodemographic factors, the research highlights that living near poor drainage systems and contact with infected individuals are the most frequently cited lifestyle factors (54.05% and 53.92%, respectively) associated with skin diseases. This underscores the importance of environmental sanitation and hygiene in maintaining skin health, consistent with previous studies emphasizing the need for improvements in these areas to reduce the burden of skin diseases [31–33, 44, 46]. Among environmental factors, exposure to sunlight (81.56%) and extremely heated weather (78.62%) emerge as the most reported factors. These observations align with existing literature which identified sunlight as a significant trigger for skin diseases [33, 46]. Further evidence demonstrates that heated weather plays a crucial role in exacerbating skin conditions [33, 45–47]. The dietary analysis identifies specific food items associated with skin diseases in Bangladesh, with beef (65.36%), hilsa (48.60%), and brinjal (45.53%) being the most frequently cited. These patterns align with the existing study [34]. The genetic analysis reveals significant associations between skin diseases and family history, particularly among sisters (39.59%), mothers (39.02%), and fathers (33.58%). This pattern supports existing literature on the heritability of various skin diseases and emphasizes the importance of genetic factors in skin disease susceptibility [22].

To provide a more comprehensive analysis of these relationships, PCA and subsequent PCLR analysis reveal that heritable factors are the most significant predictor for developing fungal infections. These results align with existing literature on heritable factors in this condition [55, 60]. Meanwhile, environmental factors emerged as the primary predictor for developing acne vulgaris, supporting previous research findings [57, 59].

The robustness of these findings was evaluated through goodness‐of‐fit testing and sensitivity analyses adjusting for key sociodemographic confounders. Among the associations identified in the unadjusted PCLR models, two demonstrated adequate model fit and retained statistical significance after adjustment: fungal infection with heritable factors and acne vulgaris with environmental factors. These associations persisted with minimal attenuation after adjustment, indicating that they are independent of sociodemographic characteristics. In contrast, the association between atopic dermatitis and food factors, although significant in both unadjusted and adjusted models, was excluded due to inadequate Hosmer–Lemeshow calibration (*p* = 0.01) after adjustment, indicating poor model fit despite the association strengthening numerically. The association between acne vulgaris and lifestyle factors, while significant in the unadjusted model, demonstrated poor model calibration after adjustment and was therefore excluded from the final sensitivity analysis. The association between seborrheic dermatitis and heritable factors, although significant in both unadjusted and adjusted models, was also excluded due to inadequate Hosmer–Lemeshow calibration (*p* = 0.04) after adjustment, indicating poor model fit. Similarly, all associations initially observed for psoriasis with lifestyle, environmental, food, and heritable factors, although statistically significant, were excluded due to inadequate Hosmer–Lemeshow calibration after adjustment for sociodemographic confounders, indicating poor model fit across all factor domains. This underscores the critical importance of evaluating both statistical significance and model fit in observational studies of skin disease risk factors, particularly in a sociodemographic diverse population such as Bangladesh.

### 4.1. Limitations of the Study

1. The cross‐sectional design precludes causal inference and assessment of temporal relationships between risk factors and skin disease outcomes, limiting the depth of understanding of underlying causal mechanisms.
2. The use of purposive quota sampling may introduce selection bias, as respondents were not randomly selected, potentially limiting the representativeness of the sample and the generalizability of findings beyond the studied population.
3. Geographic coverage was restricted to three divisions of Bangladesh, potentially limiting the generalizability of findings to the broader Bangladeshi population.
4. Reliance on self‐reported data may introduce biases such as misreporting, underreporting, or misclassification of skin conditions, particularly due to the use of medical terminology that may not be familiar to all participants.
5. Although key sociodemographic confounders were adjusted for in the sensitivity analyses, unmeasured or residual confounding may still be present, and potential mediating pathways between identified risk factors and skin disease development remain unexplored.
6. The use of Pearson correlation‐based PCA on binary variables, while empirically validated in this study, represents a methodological consideration that future research may wish to address through alternative approaches such as tetrachoric correlation‐based PCA.

## 5. Conclusions

This comprehensive study provides valuable insights into the prevalence and determinants of skin diseases in Bangladesh, revealing a considerably higher prevalence (61.80%) than previously documented. Specifically, our findings demonstrate that atopic dermatitis is the most prevalent condition (72.07%), followed by fungal infection (26.90%), acne vulgaris (20.74%), seborrheic dermatitis (13.55%), and psoriasis (8.21%). Moreover, the study successfully identifies multiple significant associations between various skin conditions and sociodemographic factors, environmental conditions, lifestyle choices, dietary patterns, and genetic predispositions.

In examining these relationships more closely through PCA and subsequent logistic regression, we conducted rigorous goodness‐of‐fit testing in both unadjusted and adjusted models to evaluate model calibration for all identified associations. Only two associations demonstrated adequate model fit in the adjusted models: heritable factors with fungal infection (unadjusted OR: 1.34, 95% CI: 1.08–1.66) and environmental factors with acne vulgaris (unadjusted OR: 1.45, 95% CI: 1.16–1.80). These two validated associations were carried forward for sensitivity analysis in Figure 4, which displays both their unadjusted and adjusted odds ratios.

Sensitivity analyses adjusting for key sociodemographic confounders confirmed the robustness of these two associations, with both findings remaining significant after adjustment for age, sex, division, and disease‐specific confounders. For fungal infection, the association with heritable factors persisted after adjustment (adjusted OR: 1.37, 95% CI: 1.09–1.74). The association between acne vulgaris and environmental factors remained significant (adjusted OR: 1.32, 95% CI: 1.05–1.67). The association between atopic dermatitis and food factors, although statistically significant in both unadjusted and adjusted models, was excluded due to inadequate Hosmer–Lemeshow calibration (*p* = 0.01) after adjustment, indicating poor model fit despite numerical strengthening. The association between acne vulgaris and lifestyle factors, while significant in the unadjusted model, demonstrated poor model calibration after adjustment and was therefore not considered a robust finding. The association between seborrheic dermatitis and heritable factors, although significant in both unadjusted and adjusted models, was excluded due to inadequate Hosmer–Lemeshow calibration (*p* = 0.04) after adjustment. Similarly, all associations initially observed for psoriasis with lifestyle, environmental, food, and heritable factors, although statistically significant, were excluded due to inadequate Hosmer–Lemeshow calibration after adjustment, indicating poor model fit across all factor domains. This underscores the critical importance of evaluating both statistical significance and model calibration in observational studies of skin disease risk factors, as statistical significance alone is insufficient to establish the reliability of observed associations.

These findings carry important practical implications for public health interventions in Bangladesh. The identification of heritable factors as significant predictors of fungal infection underscores the need for family‐based screening and early preventive interventions, while the association between environmental factors and acne vulgaris highlights the importance of environmental regulation and public awareness campaigns targeting pollution and heat exposure. Given the limited access to dermatological care across Bangladesh, particularly in rural and underserved areas, telemedicine represents a promising avenue for bridging the gap between diagnosis and treatment. Telemedicine platforms could facilitate early identification of skin conditions, enable remote consultation with dermatologists, and support the delivery of targeted interventions based on the risk factor profiles identified in this study, ultimately reducing the burden of skin diseases at the population level.

Despite these significant contributions, the study has several limitations. The cross‐sectional design precludes causal inference and assessment of temporal relationships between risk factors and outcomes. The reliance on self‐reported diagnoses without clinical verification may have resulted in misclassification of skin conditions, particularly given the use of medical terminology that may not be familiar to all participants. Geographic coverage was limited to three divisions, potentially limiting generalizability to the entire Bangladeshi population. Additionally, while we adjusted for measured sociodemographic confounders, unmeasured or residual confounding may still be present. Furthermore, the use of Pearson correlation‐based PCA on binary variables, while empirically validated in this study, represents a methodological consideration that future research may wish to address through alternative approaches such as tetrachoric correlation‐based PCA. Future research should address these limitations through longitudinal studies with clinical confirmation of diagnoses, broader geographic sampling across all divisions of Bangladesh, objective measures of exposure assessment, and investigation of potential mediating pathways between identified risk factors and skin disease development.

## Funding

This study was supported by the SUST Research Center, PS/2022/2/34.

## Disclosure

The current study forms part of the project titled “Identifying the factors related to skin diseases in Bangladesh: A cross‐sectional study”.

## Conflicts of Interest

The authors declare no conflicts of interest.

## Supporting Information

Additional supporting information can be found online in the Supporting Information section.

## Supporting information


**Supporting Information** Additional supporting information includes the questionnaire and informed consent form used in this study.

## Data Availability

Study data, related code, and materials can be obtained from the corresponding author if the request is deemed reasonable.
