# Supplementary material for: Risk Factors Associated With Major Skin Diseases: A Cross‐Sectional Study From Bangladesh
Source: Glob Health Epidemiol Genom. 2026 Jun 20;2026:8995557. doi: 10.1155/ghe3/8995557 (PMC13283032; doi:10.1155/ghe3/8995557)
Supplement: Supplementary file 1 — Supporting Information Additional supporting information includes the questionnaire and informed consent form used in this study. [file GHE3-2026-8995557-s001.docx]

**Survey on Skin Disease**

The questionnaire is developed for a part of the project titled as “Identifying the Factors Related to Skin Diseases in Bangladesh: A Cross-sectional Study”. This project is funded by SUST Research Center, SUST, Sylhet, Bangladesh. **The information provided by you in this questionnaire will be used for research purposes only. It will not be used in a manner which would allow identification of your individual responses**.

Information having asterisk marks (*) allow multiple answers

|  | | ID |  | |
| --- | --- | --- | --- | --- |
|  | | Gender | Female |  |
|  |  |  | Male |  |
|  | | Division | Dhaka |  |
|  |  |  | Rangpur |  |
|  |  |  | Chattogram |  |
|  | | Age (in years) |  |  |
|  | | Education |  |  |
|  | | Profession |  |  |
|  | | Marital status | Unmarried |  |
|  |  |  | Married |  |
|  | | Source of usable water | Tubewell |  |
|  |  |  | Supply |  |
|  |  |  | Submersible pump |  |
|  | | Type of the house | Flat |  |
|  |  |  | Building |  |
|  |  |  | Half-building |  |
|  |  |  | Tin/Slum |  |
|  | | Presence of skin disease during survey | Yes |  |
|  |  |  | No |  |
| If ‘**Yes**’ in the 11^th^ information, proceed further! | | | | |
|  | | Type of skin disease* | Acne vulgaris | ☐ |
|  |  |  | Atopic dermatitis | ☐ |
|  |  |  | Fungal infections | ☐ |
|  |  |  | Seborrheic dermatitis | ☐ |
|  |  |  | Psoriasis | ☐ |
| Please mention the presence/absence of certain lifestyle factor(s)*  Ticking a box will indicate the presence of that certain factor | | | | |
|  | | Anxiety or depression | |  |
|  | | Sudden anger | |  |
|  | | Showering too little | |  |
|  | | Excessive sweating | |  |
|  | | Wearing unclean clothes | |  |
|  | | Weakened immune system | |  |
|  | Use illicit drugs or alcohol | | |  |
|  | Contact with infected individuals | | |  |
|  | Affected by insect bites | | |  |
|  | Use cosmetics | | |  |
|  | Larger family size (extended family) | | |  |
|  | Living near poor drainage system | | |  |
| Please mention the presence/absence of certain environmental factor(s)*  Ticking a box will indicate the presence of that certain factor | | | | |
|  | Living in an area with lack of air-conditioning | | |  |
|  | Living in a room under thermal discomfort | | |  |
|  | Exposure to heavy metal in indoor air | | |  |
|  | Exposure to sunlight in the room | | |  |
|  | Dust Mites | | |  |
|  | Extremely heated weather | | |  |
|  | Cold weather | | |  |
|  | Humidity | | |  |
|  | Polluted air | | |  |
|  | Polluted water | | |  |
|  | Climate change | | |  |
| Please mention the consumption of certain food item(s)*  Ticking a box will indicate that you consume that certain food | | | | |
|  | Hilsa | | |  |
|  | Shrimp | | |  |
|  | Brinjal | | |  |
|  | Spinach | | |  |
|  | Arum | | |  |
|  | Lentil | | |  |
|  | Corn | | |  |
|  | Beef | | |  |
|  | Mutton | | |  |
|  | Duck | | |  |
|  | Pineapple | | |  |
|  | Chicken’s egg | | |  |
|  | Duck’s egg | | |  |
| Please mention the presence/absence of heritable factor(s)*  Ticking a box will indicate that certain person has/had skin disease(s) | | | | |
|  | Father | | |  |
|  | Mother | | |  |
|  | Brother | | |  |
|  | Sister | | |  |
|  | Grandfather | | |  |
|  | Grandmother | | |  |
|  | Paternal Cousin | | |  |
|  | Maternal Cousin | | |  |
|  | Husband | | |  |
|  | Wife | | |  |
|  | Son | | |  |
|  | Daughter | | |  |
|  | None | | |  |
